# Ag Nanoparticles Decorated ZnO Nanorods as Multifunctional SERS Substrates for Ultrasensitive Detection and Catalytic Degradation of Rhodamine B

**DOI:** 10.3390/nano12142394

**Published:** 2022-07-13

**Authors:** Xingang Chen, Lei Zhu, Zhipeng Ma, Meilin Wang, Rui Zhao, Yueyue Zou, Yijie Fan

**Affiliations:** 1 School of Electrical and Electronic Engineering, Chongqing University of Technology, Chongqing 400054, China; chenxingang@cqut.edu.cn (X.C.); wangmeilin@stu.cqut.edu.cn (M.W.); zhaorui@stu.cqut.edu.cn (R.Z.); zouyueyue@stu.cqut.edu.cn (Y.Z.); fanyijie@stu.cqut.edu.cn (Y.F.); 2 Chongqing Engineering Research Center of Energy Interconnection, Chongqing 400054, China

**Keywords:** SERS, organic pollutants, ZnO/Ag, sensitivity, photocatalytic

## Abstract

Industrial wastewater containing large amounts of organic pollutants is a severe threat to the environment and human health. Thus, the rapid detection and removal of these pollutants from wastewater are essential to protect public health and the ecological environment. In this study, a multifunctional and reusable surface-enhanced Raman scattering (SERS) substrate by growing Ag nanoparticles (NPs) on ZnO nanorods (NRs) was produced for detecting and degrading Rhodamine B (RhB) dye. The ZnO/Ag substrate exhibited excellent sensitivity, and the limit of detection (LOD) for RhB was as low as 10^−11^ M. Furthermore, the SERS substrate could efficiently degrade RhB, with a degradation efficiency of nearly 100% within 150 min. Moreover, it retained good SERS activity after multiple repeated uses. The interaction between Ag NPs, ZnO, and RhB was further investigated, and the mechanism of SERS and photocatalysis was proposed. The as-prepared ZnO/Ag composite structure could be highly applicable as a multifunctional SERS substrate for the rapid detection and photocatalytic degradation of trace amounts of organic pollutants in water.

## 1. Introduction

With the rapid development of global industrialisation, the discharge of wastewater containing large amounts of organic pollutants is a severe threat to the environment and human health [1,2,3]. For example, Rhodamine B (RhB) is a common organic pollutant in wastewaters. It is often used as a dye in food, textiles, and leather industries because of its bright colour. Studies have shown that RhB is a carcinogen that accumulates in living organisms and seriously harms health. The maximum residue limit of RhB in food is 500 µg/kg according to the European Union standard. Some European and American countries have severely restricted the use of this substance [4,5,6]. The organic pollutants in water in trace or even ultra-trace can have a detrimental effect, so it is particularly critical to develop a rapid, simple, and highly sensitive technique for detecting and degrading organic pollutants in water.

Compared with traditional liquid chromatography-mass spectrometry (LC-MS) [7] and high-performance liquid chromatography (HPLC) [8], surface-enhanced Raman scattering (SERS) is a promising ultra-trace detection technology with numerous advantages, such as being a fast and straightforward method, having high sensitivity, and is non-destructive. These properties make it widely used in various fields such as analytical chemistry, life science, and food safety, and it has good prospects for the rapid detection of organic pollutants [9,10,11,12].

The enhancement of the Raman signal in SERS detection depends on the choice of the substrate material. The noble metal nanostructures’ local surface plasmon resonance (LSPR) frequencies lie in the excitation wavelength range frequently used in Raman spectroscopy. Therefore, these materials are often used as SERS substrates to detect target molecules [13,14]. However, these noble metal SERS substrates have some drawbacks in actual usage: (1) Noble metal nanoparticles are easily agglomerated and have relatively low stability. (2) The substrates are not self-cleaning and cannot be reused [15,16]. One factor to consider when detecting organic pollutants in real life is that trace levels of organic pollutants are constantly present in water sources and animals and could seriously harm human health through the accumulation of the food chain. Improvements and innovations in the functionalisation of nano-substrates are essential for applying SERS technology to detect organic pollutants. Efficient SERS substrates require not only high sensitivity and good stability but also good reproducibility and effective degradation of organic pollutants [17,18,19].

To date, the degradation of organic pollutants from wastewater using semiconductor materials with excellent photocatalytic properties (TiO_2_, ZnO, etc.) has been proven to be an efficient and green approach [20,21]. However, the low conduction band (CB) electron density and weak LSPR effect prevent semiconductor materials from using SERS substrates to detect trace organic pollutants [22,23]. Fortunately, the incorporation of semiconductors and noble metals provides a new pathway to prepare highly efficient SERS substrates. The combination of the two facilitates the integration of the LSPR effect of noble metal with the charge transfer (CT) phenomenon at the metal-semiconductor interface, and the synergistic effect could significantly improve the effectiveness of SERS detection [24,25,26,27]. In addition, due to the Schottky barrier formed between the semiconductor and noble metal, the electron-hole recombination process of the composite structure is effectively slowed down, which is beneficial to further improve the photocatalytic efficiency [28,29,30,31]. ZnO is inexpensive, biocompatible, chemically stable, and rich in morphological structure among the many semiconductors. The noble metal/ZnO hybrid structures are ideal materials for achieving a good SERS effect [32,33]. Additionally, the content and uniform distribution of noble metals on the ZnO surface are important and the formation of dense “hot spots” is beneficial to improving detection effects.

In this study, we synthesized a substrate with SERS detection and photocatalytic degradation by depositing Ag nanoparticles (NPs) on ZnO nanorods (NRs) and optimised the content of Ag and uniform distribution on the surface of ZnO by varying the amount of Ag NO_3_ added. The substrate exhibited high sensitivity to the model pollutant (RhB) due to the strong LSPR effect of the noble metal in combination with the CT mechanism at the contact surface of the metal-semiconductor heterojunction, and the limit of detection (LOD) for RhB was 10^−11^ M. Additionally, the as-prepared ZnO/Ag substrate exhibited good photocatalytic degradation properties under visible light irradiation and maintained excellent self-cleaning properties after several cycles. The substrate had high sensitivity, good stability, and high reproducibility, which could quickly and easily detect trace amounts of organic pollutants in water and realise their rapid photocatalytic degradation.

## 2. Materials and Methods

### 2.1. Materials

Rhodamine B (RhB, 98%), and silver nitrate (AgNO_3_, 99.9%) were purchased from Cologne Chemical Co., Ltd. (Chengdu, China). Trisodium citrate (C_6_H_5_Na_3_O_7_, 99.9%) and polyvinylpyrrolidone (PVP, 99.9%, Mw ≈ 58,000) were purchased from Aladdin Reagent Co., Ltd. (Shanghai, China). Zinc oxide nanorods were purchased from XFNANO Materials Technology Co., Ltd. (Nanjing, China). Millipore ultrapure water (>18 MΩ) and anhydrous ethanol were used in all experiments.

### 2.2. Characterization

X-ray diffraction (XRD) patterns were acquired by the PANalytical X’pert PRO diffractometer (PANalytical B.V., Almelo, The Netherlands). The micromorphology of the substrates was observed by FESEM (ΣIGMA, Zeiss, Jena, Germany). The high-resolution transmission electron microscopy was analyzed by TEM (JEM-2100F, Japan Electronics Co., Ltd., Tokyo, Japan). X-ray photoelectron spectroscopy (ESCALAB 250Xi, Waltham, MA, USA) was used to analyze the crystal surface composition of the substrates. SERS measurements were conducted with a commercial micro-Raman spectrometer (Ahalp300, WITec, Ulm, Germany). The laser wavelength was 532 nm, and the power was 0.5 mW. A 100× objective was used, and the integral time of the spectrometer was set at 1 s.

### 2.3. Synthesis of ZnO/Ag

To uniformly deposit Ag NPs on ZnO NRs, 80 mg of ZnO NRs was ultrasonically dispersed in 100 mL of water, and then 150 mg of PVP and a small amount of AgNO_3_ were added through stirring. After heating the mixed solution to 100 °C, 2 mL Trisodium citrate solution (1 wt%) was added and reacted for 1 h through stirring. At the end of the reaction, the black product was collected by centrifugation at 10,000 r/min and washed 3 times with water and anhydrous ethanol. Finally, the black product was dried at 60 °C in a vacuum drying oven.

The content of Ag on ZnO NRs had a significant influence on the detection effect. By changing the amount of applied AgNO_3_ (10 mg, 20 mg, 30 mg), the composite structures with different amounts of Ag were obtained and named ZnO/Ag1, ZnO/Ag2, and ZnO/Ag3.

### 2.4. SERS Detection

**Figure 1:**
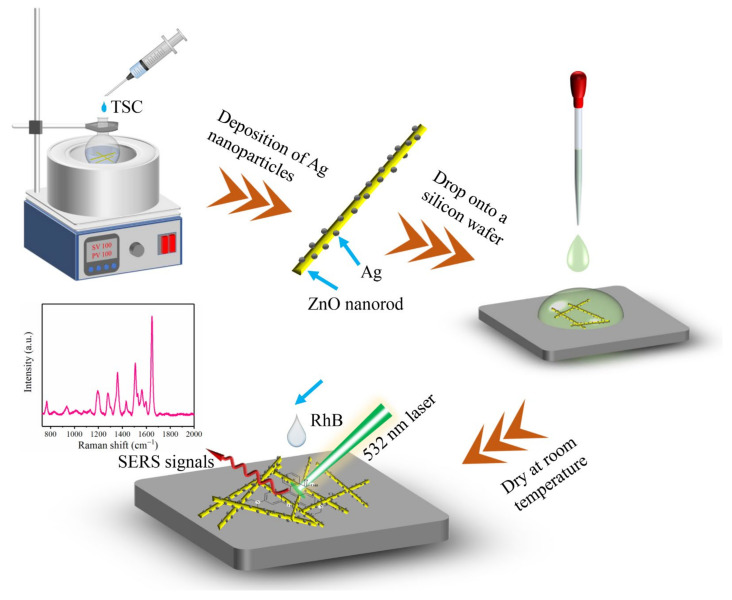
The preparation of the SERS substrate and SERS detection process.

Silicon wafers of 1 cm × 0.5 cm in size were washed alternately with water and anhydrous ethanol for 10 min and dried at room temperature for later use. In total, 10 mg ZnO/Ag powder was ultrasonically dispersed in 2 mL anhydrous ethanol, and then 20 µL ZnO/Ag suspension was dropped onto clean silicon wafers to prepare the SERS substrates. Then, 10 µL of various concentrations of RhB aqueous solution (10^−4^ M–10^−11^ M) was dropped onto the SERS substrates and dried at room temperature for SERS detection. The preparation of the SERS substrate and SERS detection process is presented in Figure 1. After SERS detection, the substrate was immersed in water (4 mL) and irradiated under visible light (Xe lamp, λ > 420 nm). After irradiation, the substrate was rinsed with water, dried at room temperature, and re-tested for SERS.

## 3. Results

### 3.1. Characterisation of ZnO/Ag

**Figure 2:**
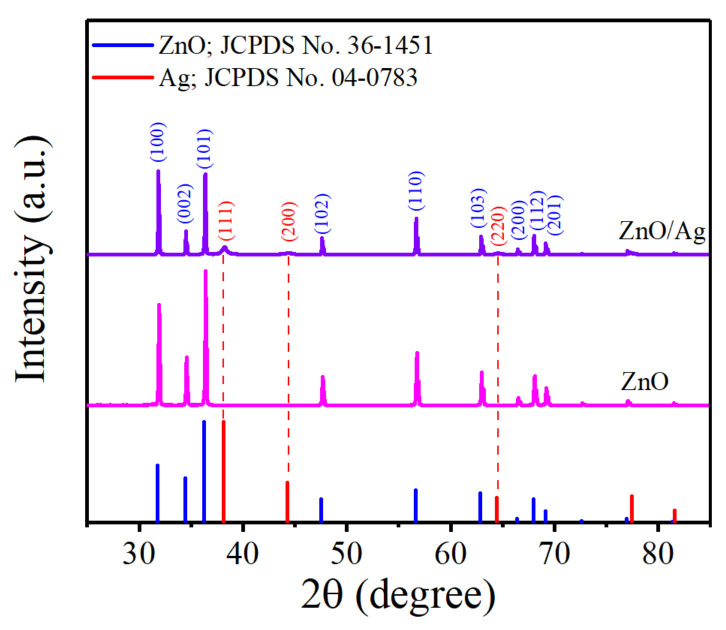
XRD patterns of ZnO and ZnO/Ag.

The crystal structure information of the prepared products was measured using XRD. As shown in Figure 2, the peak positions of pure ZnO NRs are consistent with the standard XRD data of ZnO (JCPDS Card No. 36-1451), corresponding to the (100), (002), (101), (102), (110), (103), (220), (112), and (201) crystal planes. The diffraction peaks of ZnO NRs are sharp, indicating that ZnO NRs have high crystallinity. For the ZnO/Ag composite, in addition to the peaks of ZnO, the diffraction peaks of Ag can be seen, corresponding to the (111), (200), and (220) crystal planes of Ag (JCPDS card No. 04-0783). No other spurious peaks are observed in the prepared structure, proving that the complex is not polluted during the preparation process and has high purity.

**Figure 3:**
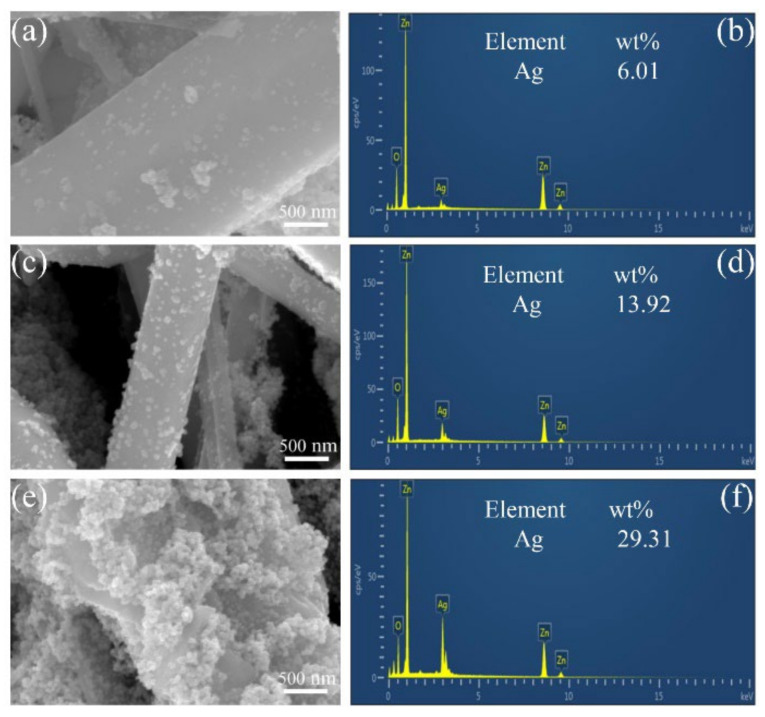
(**a**,**c**,**e**) SEM images of ZnO/Ag. (**b**,**d**,**f**) EDS spectra of ZnO/Ag.

Figure 3 shows the FESEM images and EDS spectra of ZnO/Ag composite with different Ag contents. It can be observed that the amount of Ag NPs on ZnO NRs increases with the rise in the amount of AgNO_3_. As shown in Figure 3a, when the amount of AgNO_3_ is 10 mg, fewer Ag NPs are deposited on the ZnO NRs and the EDS spectrum shows that the surface content of Ag is 6.01% (Figure 3b). The nano-gaps between Ag NPs could contribute significantly to the SERS effect. The nano-gaps between Ag NPs in the ZnO/Ag1 composite is greater than 50 nm, preventing the creation of a high-density “hot spot”. When the amount of AgNO_3_ is increased to 20 mg, the Ag NPs are deposited more uniformly on the ZnO NRs, and the density of Ag NPs is higher than in Figure 3a. According to the EDS spectrum, the surface content of Ag on ZnO NRs increased to 13.92% (Figure 3d). The gaps between the Ag NPs are reduced to 10–20 nm, and the smaller nano-gaps help to form more “hot spots” and improve the SERS enhancement (Figure 3c). When AgNO_3_ is increased to 30 mg, more Ag NPs are deposited on the ZnO NRs, and adjacent Ag NPs are stacked together and wrapped around the ZnO NRs surface (Figure 3e). The EDS spectrum shows that the surface content of Ag increased to 29.31% (Figure 3f), and the agglomeration phenomenon may lead to the reduction of the gaps between nanoparticles, which is not beneficial to SERS enhancement.

**Figure 4:**
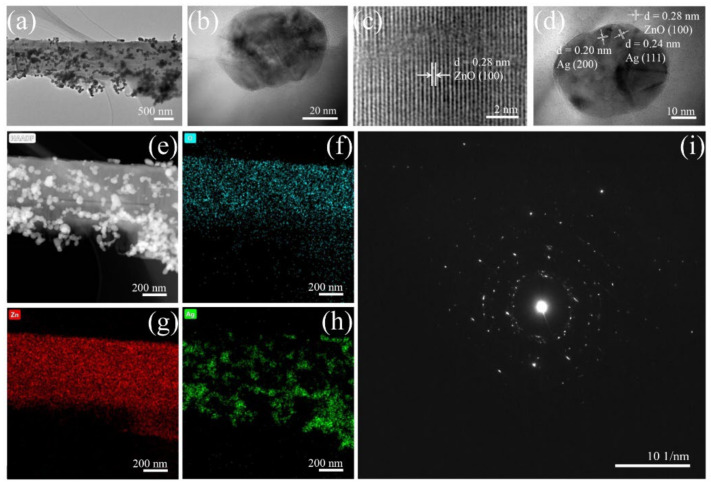
(**a**) TEM micrograph of ZnO/Ag2. (**b**–**d**) HRTEM micrograph of ZnO/Ag2. (**e**) STEM micrograph of ZnO/Ag2. (**f**–**h**) Elemental mappings of O, Zn, and Ag. (**i**) SAED pattern of ZnO/Ag2.

The ZnO/Ag2 composites microstructure is analysed in more detail using HRTEM. As shown in Figure 4a, a large amount of Ag NPs is deposited on the surface of the ZnO NRs with a particle size of approximately 50 nm. The high-resolution image (Figure 4b) shows the formation of the ZnO/Ag interface, which offers the possibility of charge transfer between the two, contributing to the enhanced Raman signal when measured by SERS. In Figure 4c, a clear lattice fringe spacing of 0.28 nm can be observed, corresponding to the ZnO (100) crystal plane. The lattice spacings of Ag NPs in the composite structure are 0.24 nm and 0.20 nm, corresponding to the (111) and (200) crystal planes of cubic Ag, respectively (Figure 4d). In the corresponding selected area electron diffraction (SAED) pattern (Figure 4i), the Ag NPs in the ZnO/Ag2 composite have a polycrystalline structure with concentric diffraction rings, further confirmed as the (111), (200), (220), and (311) crystal faces of Ag, and this is consistent with the XRD data. The TEM mapping confirms the presence of O, Ag, and Zn, with Ag elements distributed on ZnO, representing the successful synthesis of the ZnO/Ag composite structure (Figure 4e–h).

**Figure 5:**
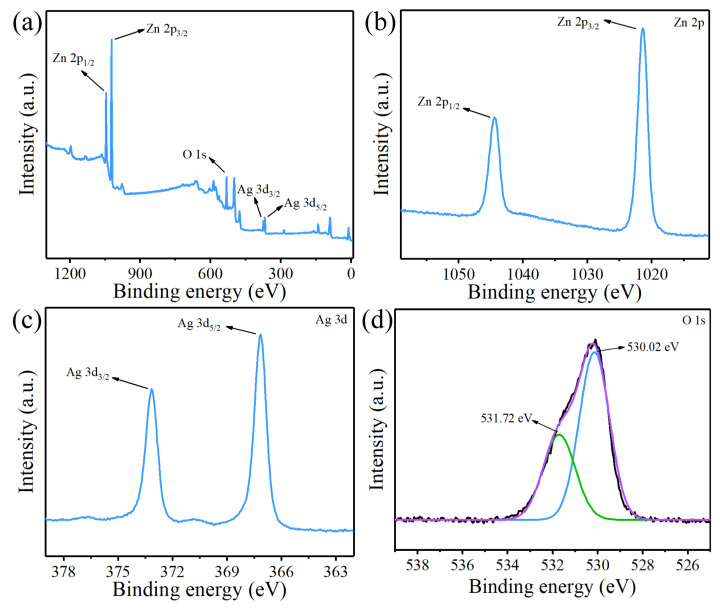
(**a**) XPS of ZnO/Ag2. (**b**–**d**) High-resolution XPS spectra of Zn 2p, Ag 3d, and O 1s.

The surface composition and chemical state of the composite structure were measured by XPS. As shown in Figure 5a, the XPS spectra prove the presence of the Zn, O, and Ag. Figure 5b shows the high-resolution XPS spectra of Zn 2p. The binding energy peaks at 1021.4 and 1044.4 eV correspond to Zn 2p_3/2_ and Zn 2p_1/2_, and this indicates that the Zn in the structure is in the form of Zn^2+^. In the high-resolution XPS spectra of Ag 3d (Figure 5c), the binding energy peaks at 373.15 and 367.15 eV correspond to Ag 3d_3/2_ and Ag 3d_5/2_, respectively. The splitting energy between Ag 3d_5/2_ and Ag 3d_3/2_ is 6.0 eV, demonstrating that the Ag element in the composite is present in the Ag^0^ state. Compared with the binding energies of Ag 3d_3/2_ and Ag 3d_5/2_ of pure Ag (374.3 and 368.3 eV, respectively), the peaks of Ag 3d in the composite structure are negatively shifted, which may be due to the strong interaction between ZnO and Ag and the CT phenomenon in the composite. The high-resolution XPS spectrum of the composite structure O 1s is shown in Figure 5d. The O 1s can be split into two Gaussian peaks. The peak at 530.02 eV corresponds to lattice oxygen (O_L_), and the peak at 531.72 eV corresponds to surface hydroxyl oxygen (O_H_) induced by structural defects. These oxygen groups can efficiently inhibit the recombination of electron-hole pairs and further enhance the efficiency of CT [34,35].

### 3.2. SERS Detection of Rhodamine B

**Figure 6:**
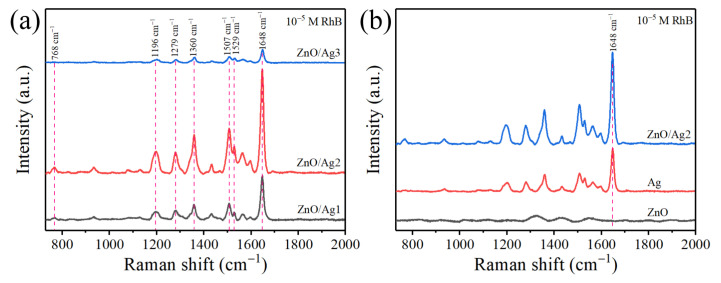
(**a**) Comparison of the SERS spectra of 10^−5^ M RhB on ZnO/Ag substrates with different Ag contents. (**b**) SERS spectra on different substrates with 10^−5^ M RhB.

To test the SERS activity of ZnO/Ag composite structures with different amounts of silver loading, RhB of 10^−5^ M was used as the probe molecule to evaluate the performance of the substrates. As shown in Figure 6a, with the increase of Ag loading, the SERS intensity of the probe molecule shows a trend of first increasing and then decreasing, and the ZnO/Ag2 composite structure shows the best SERS activity. SERS activity is usually closely related to the number of “hot spots”. When less AgNO_3_ is added during the synthesis, fewer Ag NPs are deposited on the ZnO NRs, and the larger nano-gaps do not form dense “hot spots”, resulting in poor SERS enhancement. When more AgNO_3_ is added, many Ag NPs agglomerate together, leading to the disappearance of many nano-gaps and a sharp decrease in the SERS activity. The appropriate amount of AgNO_3_ addition facilitates the uniform deposition of Ag NPs on the ZnO NRs. Furthermore, the size and density of the interstitial spaces are relatively uniform, which is beneficial for the generation of highly dense “hot spots” and the enhancement of Raman scattering intensity. The detection results are consistent with the phenomenon observed in the SEM images. Therefore, the ZnO/Ag2 composite is chosen as the optimal SERS substrate for subsequent investigations.

To evaluate the SERS performance of ZnO bound to Ag, SERS spectra of 10^−5^ M RhB solution adsorbed on ZnO, Ag, and ZnO/Ag2 were compared. According to Figure 6b, no characteristic peaks of RhB are observed in the SERS spectra of the ZnO substrate. This is because ZnO could not generate LSPR under the action of a laser, resulting in negligible SERS activity. However, due to the LSPR effect of Ag in visible light, the SERS spectra measured on a pure Ag substrate show characteristic peaks of RhB at 768, 1196, 1279, 1360, 1507, 1529, and 1648 cm^−1^. The vibrational modes corresponding to the characteristic peaks are summarised in Table 1 [36]. Because the Raman vibration at 1648 cm^−1^ is the strongest, the SERS performance is assessed using the Raman intensity at 1648 cm^−1^. Compared with pure Ag substrate, the characteristic peak intensity of RhB adsorbed on ZnO/Ag2 substrate is significantly enhanced. After ZnO is combined with Ag, good SERS sensitivity improves the detection limit of RhB and realises the detection of RhB with a lower concentration.

**Figure 7:**
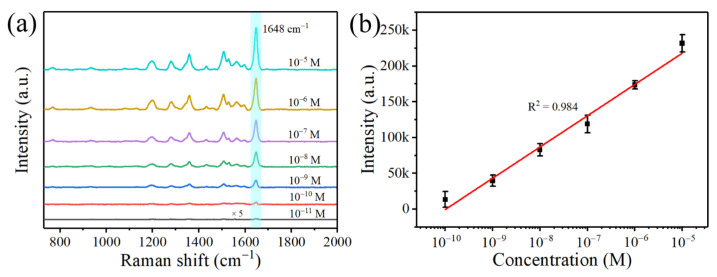
(**a**) SERS spectra of RhB at a concentration of 10^−5^ M to 10^−11^ M. (**b**) The logarithm of RhB concentration and Raman intensity plot at peak 1648 cm^−1^.

The sensitivity of the substrate was further examined using different concentrations of RhB. As shown in Figure 7a, the intensity of the Raman characteristic peaks decreases in parallel with the concentration of RhB. Moreover, the characteristic peak at 1648 cm^−1^ can be identified even at a concentration of 10^−11^ M. Using 1648 cm^−1^ Raman intensity as the standard for quantitative analysis, Figure 7b shows an excellent linear response between SERS intensity and the logarithm of RhB concentration, with a linear concentration range of 10^−10^ M to 10^−5^ M. The regression equation is *I* = 437,946.71 + 43,730.02 × Log*C*, and the correlation coefficient *R*^2^ is 0.984. The enhancement factor (EF) is an essential indicator for quantifying the performance of the substrate, and the EF for SERS detection is calculated according to the following equation:

(1) $$EF=\frac{I_{\mathrm{SERS}}}{I_{\mathrm{BARE}}}\times \frac{C_{\mathrm{BARE}}}{C_{\mathrm{SERS}}}$$

where $I_{\mathrm{SERS}}$ and $I_{\mathrm{BARE}}$ are the intensities of the Raman signals acquired from the SERS substrate and the Si substrate ($I_{\mathrm{SERS}}$ = 886 a.u., $I_{\mathrm{BARE}}$ = 962 a.u., respectively). $C_{\mathrm{SERS}}$ and $C_{\mathrm{BARE}}$ are the concentrations of the RhB solution corresponding to the SERS detection and the normal Raman detection ($C_{\mathrm{SERS}}$ = 10^−11^ M, $C_{\mathrm{BARE}}$ = 10^−2^ M, respectively). Thus, the calculated EF is 9.2 × 10^8^, indicating a high level of SERS performance of the ZnO/Ag2 substrate. As shown in Table 2, we compare the EF with that of other ZnO/Ag materials. We find that our prepared ZnO/Ag nanorods have a higher EF, which is beneficial for the detection of RhB at a lower concentration.

**Figure 8:**
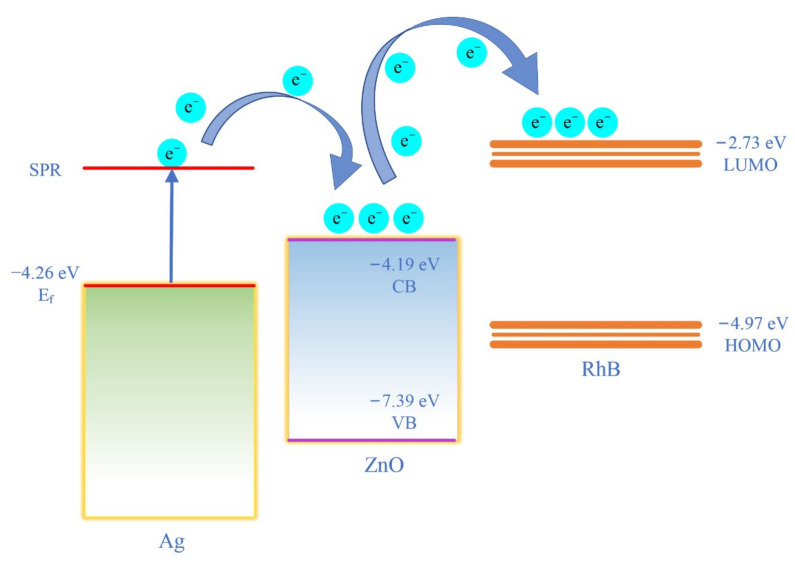
Schematic of CT mechanism between Ag, ZnO, and RhB.

Most of the previous studies have focused on demonstrating the enhanced performance of SERS substrates and have not fully explained the mechanisms associated with high-performance SERS substrates. Zhang et al. reported good test results of Ag NPs deposited on flower-like ZnO as a SERS substrate. They suggested that the high-intensity electromagnetic field formed between the Ag NPs and the chemical enhancement properties of the ZnO nanoparticles were the main reasons for the SERS enhancement [45]. Shan et al. suggested that the excellent SERS enhancement of Ag NPs deposited on wheat-like ZnO nanoarrays as a substrate was due to a combination of electromagnetic and chemical enhancement. However, they do not indicate a specific chemical enhancement mechanism [46]. For a better understanding of LSPR and the influence of CT mechanism on SERS, combined with the previous characterisations and testing results, we suggest that the great SERS performance of ZnO/Ag composite structure may be due to the following reasons: (1) More Ag NPs are adsorbed on the surface of ZnO NRs, and the dense nanogap is beneficial to the formation of “hot spots”, which will increase the electric field around the probe molecule. (2) The energy difference between the Fermi energy level of Ag (−4.26 eV) and the lowest unoccupied molecular orbital (LUMO) energy level of RhB (−2.73 eV) is less than the energy provided by the 532 nm laser (2.33 eV) [37]. Thus, photogenerated electrons can be transferred directly from the Fermi energy level of Ag to the LUMO energy level of RhB when acted upon by the laser. (3) Additionally, according to research in the field of semiconductors, CT at the semiconductor-probe molecule interface plays a critical role in enhancing the SERS signal. As depicted in Figure 8, the conduction band (CB) and valence band (VB) of ZnO are −4.19 and −7.39 eV (band gap is 3.2 eV), respectively. The CB of ZnO is between the Fermi energy level of Ag (−4.26 eV) and the LUMO of RhB (−2.73 eV). Some of the photogenerated electrons excited by LSPR are transferred from the Fermi energy level of Ag to the CB of ZnO and further to the LUMO of the RhB. The CB of ZnO functions as a “bridge” for electron transfer, which will further enhance the efficiency of CT [47,48].

**Figure 9:**
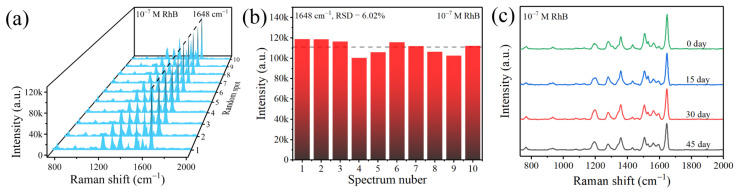
(**a**) SERS spectra obtained by randomly measuring 10 points on the ZnO/Ag2 substrate. (**b**) SERS intensity of 10^−7^ M RhB solution at 1648 cm^−1^ of the 10 SERS spectra. (**c**) SERS spectra of 10^−7^ M RhB based on ZnO/Ag2 were collected at different shelf times.

Good uniformity and oxidation resistance are essential for the practical use of the SERS substrate. To verify the uniformity of the ZnO/Ag2 substrate, 10 locations were randomly selected on the substrate adsorbed with 10^−7^ M RhB for SERS detection. Figure 9a shows the SERS spectra of 10^−7^ M RhB collected from 10 locations on the substrate, and the uniformity of the SERS signal is estimated by the relative standard deviation (RSD) of the peak intensity of 1648 cm^−1^. The RSD value is 6.02%, as shown in Figure 9b. The lower RSD value indicates that the substrate has good uniformity, favouring reproducible SERS signals from diverse locations. To verify the long-term stability of the ZnO/Ag2 substrate, SERS spectra of 10^−7^ M RhB adsorbed on the same substrate were detected every 15 days. As shown in Figure 9c, the SERS spectra do not show substantial changes during the 45 days of testing, indicating that the ZnO/Ag2 substrates have excellent oxidation resistance and can be stored at room temperature for long periods.

**Figure 10:**
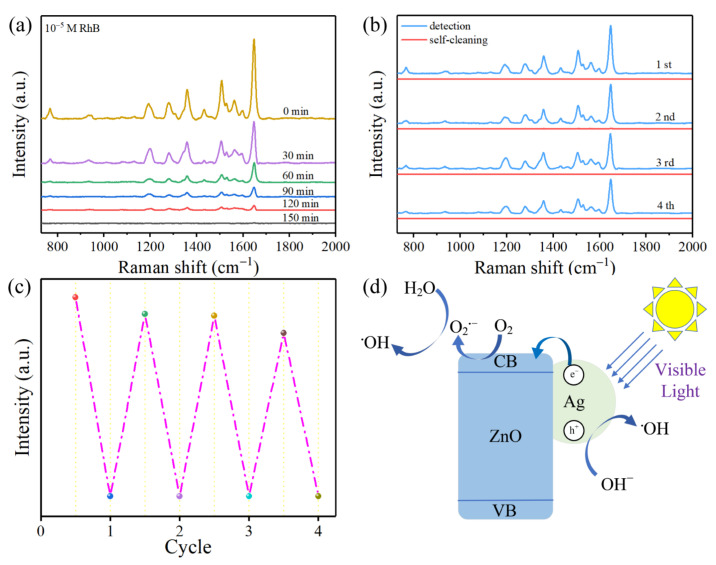
(**a**) SERS spectra of the degradation of 10^−^^5^ M RhB. (**b**) SERS spectra of 10^−5^ M RhB before and after the self-cleaning test. (**c**) Raman intensity at 1648 cm^−1^ in 4-cycle detection of 10^−5^ M RhB on the substrate. (**d**) Schematic diagram of the photocatalytic process.

Based on the photocatalytic properties of ZnO/Ag2, the degradation effect of RhB by the substrate under visible light irradiation was further analysed. As seen in Figure 10a, the SERS signal intensity of 10^−5^ M RhB gradually decreases with the increase of the irradiation time. After 150 min of irradiation, the SERS signal of RhB almost completely disappears. However, after re-dropping the same concentration of RhB, the SERS intensity regains. We reused the substrate four times (waiting for RhB to degrade and then re-dropping RhB for SERS detection, repeating back and forth four times), corresponding to the four cycles in Figure 10b, respectively, and the substrate maintains good SERS activity after four cycles. The results show that the substrate can be reused at least four times, which has a certain significance to reduce the commercial cost of the substrate. Figure 10c shows the variation of the intensity of the 1648 cm^−1^ in four cycles. The intensity of the 1648 cm^−1^ decreases slightly as the number of cycles increases, related to the decrease of the “hot spot” and the adsorption capacity. In general, ZnO/Ag2 exhibits good self-cleaning properties under visible light irradiation and retains a large part of SERS activity after several cycles.

The mechanism of photocatalytic degradation of RhB by ZnO/Ag2 under visible light irradiation is shown in Figure 10d. Under irradiation, the free electrons on the surface of Ag NPs oscillate collectively, inducing LSPR and thus the generation of electron-hole pairs. Since the energy of visible light is higher than the energy difference between the Fermi energy level of Ag (−4.26 eV) and the CB of ZnO (−4.19 eV), electrons are quickly injected into the CB of ZnO. The electrons entering ZnO react with dissolved oxygen in the water to form superoxide radical anions ($O_{2}{}^{\cdot -}$), which further react with $H_{2}O$ to form hydroxyl radicals (${}^{\cdot }OH$) in the action of visible light. In addition, the interaction between the remaining holes in Ag and the hydroxyl groups ($OH^{-}$) on the surface will also produce hydroxyl radicals (${}^{\cdot }OH$). The hydroxyl radicals (${}^{\cdot }OH$) generated are highly oxidative and further degrade RhB to $CO_{2}$ and $H_{2}O$ [49,50,51]. The proposed reaction mechanism during the photocatalytic process under the action of visible light is as follows:

(2) $$Ag+h\nu \;(\mathrm{visible}\;\mathrm{light})\;\to \;h^{+}+e^{-}$$

(3) $$Ag\;(e^{-})+ZnO\to ZnO\;(e_{CB}{}^{-})+Ag\;(h^{+})$$

(4) $$Ag\;(h^{+})+OH^{-}\to Ag+{}^{\cdot }OH$$

(5) $$ZnO\;(e_{CB}{}^{-})+O_{2}\to ZnO+O_{2}{}^{\cdot -}$$

(6) $$O_{2}{}^{\cdot -}+H_{2}O\to HO_{2}{}^{\cdot }+{}^{\cdot }OH$$

(7) $$HO_{2}{}^{\cdot }+H_{2}O\to H_{2}O_{2}+{}^{\cdot }OH$$

(8) $$H_{2}O_{2}+O_{2}{}^{\cdot -}\to {}^{\cdot }OH+O_{2}+OH^{-}$$

(9) $$H_{2}O_{2}+e^{-}\to {}^{\cdot }OH+OH^{-}$$

(10) $${}^{\cdot }OH+RhB\to CO_{2}+H_{2}O$$

However, the recombination of electron-hole pairs can significantly reduce photodegradation efficiency. Compared with pure ZnO, the addition of the noble metal Ag can efficiently prevent the occurrence of the recombination phenomenon. This is because the work function of ZnO (5.2 eV) is greater than that of Ag (4.26 eV), and when the two are combined, a Schottky barrier is formed at the Ag/ZnO interface. Schottky barriers effectively prevent the recombination of photogenerated electrons with holes, extending the lifetime of photogenerated electrons and improving the efficiency of photocatalysis [52,53]. It can be seen that ZnO/Ag composite material has good self-cleaning properties when used as a SERS substrate, facilitating the rapid degradation of organic pollutants and the reuse of the substrate.

## 4. Conclusions

In this work, we deposited Ag NPs on the surface of ZnO NRs by a simple hydrothermal method to prepare a novel SERS substrate to detect and degrade of the organic pollutant RhB in water. Due to the synergistic effect of LSPR of Ag NPs and CT mechanism, the ZnO/Ag composite showed good sensitivity, and the LOD of RhB was as low as 10^−11^ M. Furthermore, we showed that the as-prepared SERS substrate has good photocatalytic activities and that RhB degradation can be completed in 150 min. After four cycles, it could still maintain most of the SERS activity and could be reused many times. These results prove that the prepared ZnO/Ag is suitable for the detection of trace organic pollutants but also shows great potential in the degradation of organic pollutants and the recycling of the substrate.

## Figures and Tables

**Table 1:** Main peaks of RhB and their assignments.

Raman Shift (cm^−^^1^)	Peak Assignment
768	xanthene ring puckering
1196	aromatic C–C stretching
1279	C–H bending
1360	stretching vibration of bridge C–C aromatic bonds
1507	aromatic C–C bending
1529	C–H stretching
1648	the symmetric bending C–C and C=C bonds of benzene ring

**Table 2:** Comparison of EF of different ZnO/Ag materials.

SERS Substrate Based on ZnO	EF	Reference
Ag@ZnO Nanorods arrays	3.87 × 10^8^	[37]
Ag@ZnO Nanorods	1.6 × 10^6^	[38]
Ag@ZnO Nanodomes	10^6^	[39]
3D-porous ZnO/Ag	2 × 10^8^	[40]
Ag/ZnO/Si	8.7 × 10^7^	[41]
Ag@ZnO Nanosphere	3.17 × 10^8^	[42]
Ag/PDA/ZnO@GMF	10^10^	[43]
3D ZnO/Ag	3.18 × 10^9^	[44]
ZnO/Ag Nanoflowers	3.41 × 10^7^	[45]
ZnO/Ag Nanorods	9.2 × 10^8^	In this work

## Data Availability

The data presented in this paper are available on request from the corresponding author.
